# Subcategorizing the Expected Value of Perfect Implementation to Identify When and Where to Invest in Implementation Initiatives

**DOI:** 10.1177/0272989X20907353

**Published:** 2020-03-05

**Authors:** Kasper Johannesen, Magnus Janzon, Tomas Jernberg, Martin Henriksson

**Affiliations:** Department of Medical and Health Sciences, Linköping University, Linköping, Sweden; Department of Cardiology and Department of Medical and Health Sciences, Linköping University, Linköping, Sweden; Department of Clinical Sciences, Karolinska Institute, Danderyd University Hospital, Stockholm, Sweden; Department of Medical and Health Sciences, Linköping University, Linköping, Sweden

**Keywords:** health care decision making, implementation strategies, value of implementation

## Abstract

**Purpose**. Clinical practice variations and low implementation of effective and cost-effective health care technologies are a key challenge for health care systems and may lead to suboptimal treatment and health loss for patients. The purpose of this work was to subcategorize the expected value of perfect implementation (EVPIM) to enable estimation of the absolute and relative value of eliminating slow, low, and delayed implementation. **Methods**. Building on the EVPIM framework, this work defines EVPIM subcategories to estimate the expected value of eliminating slow, low, or delayed implementation. The work also shows how information on regional implementation patterns can be used to estimate the value of eliminating regional implementation variation. The application of this subcategorization is illustrated by a case study of the implementation of an antiplatelet therapy for the secondary prevention after myocardial infarction in Sweden. Incremental net benefit (INB) estimates are based on published cost-effectiveness assessments and a threshold of SEK 250,000 (£22,300) per quality-adjusted life year (QALY). **Results**. In the case study, slow, low, and delayed implementation was estimated to represent 22%, 34%, and 44% of the total population EVPIM (2941 QALYs or SEK 735 million), respectively. The value of eliminating implementation variation across health care regions was estimated to 39% of total EVPIM (1138 QALYs). **Conclusion**. Subcategorizing EVPIM estimates the absolute and relative value of eliminating different parts of suboptimal implementation. By doing so, this approach could help decision makers to identify which parts of suboptimal implementation are contributing most to total EVPIM and provide the basis for assessing the cost and benefit of implementation activities that may address these in future implementation of health care interventions.

Health gains from effective and cost-effective health care technologies are realized only when they are implemented in clinical practice. Not implementing and using effective and cost-effective technologies as intended by reimbursement authorities and clinical guidelines lead to suboptimal treatment and health loss for patients. In fact, slow implementation and clinical practice variations have been identified as a key challenge for health care systems, and considerable implementation variation exists within and across countries.^1,2^ Efforts, such as decision support tools, quality registries, and financial incentives, are being employed to address this issue,^3,4^ but there is limited research regarding the costs of suboptimal implementation and its potential to inform decision makers and researchers on how best to address the problem of low and varying implementation.

Work by Fenwick et al.^5^ outlined a framework for estimating the expected value of perfect implementation (EVPIM) that estimates the value of increasing implementation from the current or expected level of implementation up to a perfect level of implementation. The EVPIM represents an upper limit to the value of improving implementation, much in line with how the expected value of perfect information (EVPI) provides an upper limit for the value of further research regarding a specific decision problem.^5,6^

Applications and extensions of the EVPIM framework have formalized and investigated the expected value of specific implementation strategies (EVSIM), estimating the value of specific (but imperfect) improvements in implementation that may be achieved by actual or hypothetical implementation strategies.^7–17^ Some of these contributions have also demonstrated how the framework can be used to estimate the value of improving implementation in specific subgroups,^10,11^ estimating the value of implementation improvements resulting from further evidence being generated,^7,12–17^ and taking into account, as well as predicting, future implementation patterns.^10–15^ These contributions have improved the applicability and versatility of the EVPIM and EVSIM framework in assessing the value of implementation strategies based on retrospective as well as predicted future implementation patterns. Despite these developments, it has not yet been demonstrated how the EVPIM framework can be used to identify the potential underlying causes and hurdles for suboptimal implementation and how EVPIM can support decision makers in addressing the challenge of suboptimal implementation.

The purpose of this work is to outline a subcategorization of EVPIM to identify the potential value of eliminating different parts of suboptimal implementation, such as slow, low, and delayed implementation. Along the lines of expected value of partial perfect information (EVPPI) when evaluating decision uncertainty,^6^ this subcategorization can identify the absolute and relative value from eliminating different parts of suboptimal implementation and thus provide decision makers with relevant information regarding where, and potentially how, to direct resources to address them. The approach is illustrated using an example from cardiovascular disease: the implementation of ticagrelor as secondary prevention after myocardial infarction (MI) in Sweden. This is followed by a general discussion of how the proposed subcategorization can be applied to past as well as predicted future implementation patterns to support decision makers in identifying when and where to invest in implementation activities.

## Methods

The EVPIM framework set out by Fenwick et al.^5^ defines the (per patient) *expected value of perfect implementation* as the value of improving implementation from the current or expected level of implementation (ρ) to a perfect level of implementation. This is formalized in equation (1), where expected incremental net benefit (INB) is defined as the expectation over some uncertain parameters θ (E_θ_INB(θ)).^5,10,11^ (INB can be defined in terms of incremental net monetary benefit [INMB] and incremental net health benefit [INHB]^18,19^: INMB=ΔE*λ−ΔC and $\mathrm{INHB}=\Delta E-\frac{\Delta C}{\lambda }$, where ΔE and ΔC represent the incremental effect and cost, respectively, and λ the cost-effectiveness threshold.)

(1) EVPIM=(1−ρ)INB

**Figure 1:**
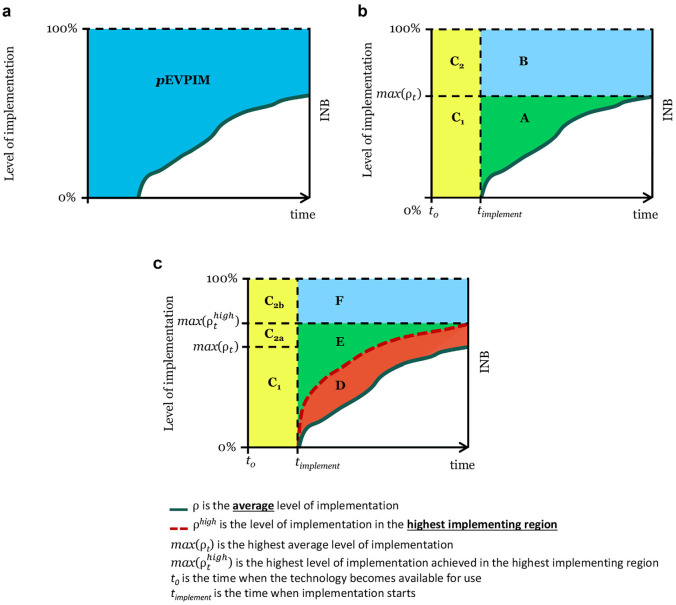
Stylised example illustrating (a) the population expected value of perfect implementation (pEVPIM); (b) the expected value of eliminating slow (A), low (B) and delayed (C = C1 + C2) implementation; and (c) the expected value of eliminating regional implementation variation (D). 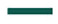
ρ is the average level of implementation. 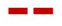
ρ^*high*^ is the level of implementation in the highest implementing region. *max*(ρ_*t*_) is the highest average level of implementation. *max*($\rho_{t}^{\mathrm{high}}$) is the highest level of implementation achieved in the highest implementing region. *t*_0_ is the time when the technology becomes available for use. *t_implement_* is the time when implementation starts.

Population EVPIM (pEVPIM) is the product of the per patient EVPIM and the number of eligible patients (*I_t_*) in each time period from when the technology becomes available (*t*_0_) until the technology loses relevance (*T*), given the discount rate *r* (equation (2) and Figure 1a).

(2) $$p\mathrm{EVPIM}=\sum_{t=0}^{T}\frac{I_{t}}{{\left(1+r\right)}^{t}}(1-\rho_{t})\mathrm{IN}B_{t}$$

The population value of a specific implementation strategy (pEVSIM) that improves implementation to ρ^*IM*^ compared to the actual/expected level of implication ρ without the implementation strategy is given by

(3) $$p\mathrm{EVSIM}=\sum_{t=0}^{T}\frac{I_{t}}{{\left(1+r\right)}^{t}}(\rho_{t}^{\mathrm{IM}}-\rho_{t})\mathrm{IN}B_{t}$$

### Subcategorizing EVPIM

In this work, we build on the previous extensions of the EVPIM framework and propose subcategorizing EVPIM to identify the value of addressing different parts of suboptimal implementation, with the aim of identifying where there is largest gain from improving implementation.

### Slow, Low, and Delayed Implementation

Population EVPIM is subcategorized into the expected value of eliminating slow, low, or delayed implementation. As illustrated in Figure 1b, we define the value of eliminating slow implementation (area A) as the value of increasing the implementation level from the current/expected level of implementation (ρ) to the highest level of implementation achieved/expected in any given time period (*max*(ρ_*t*_)), in the time after implementation is started (*t_implement_*). The value of eliminating low implementation (area B) is defined as the value of increasing implementation from the highest observed implementation level (*max*(ρ_*t*_)) to an optimal level of implementation, from the time when implementation starts. The value of eliminating delayed implementation (area C) is defined as the value of achieving a perfect level of implementation already from the time when the technology becomes available/approved/recommended for use, which in the case of pharmaceuticals in the European Union (EU) would be the time of marketing authorization approval or when recommended by health technology assessment (HTA) authorities. The value of eliminating delayed implementation (area C) is divided into the value of eliminating implementation delay without improving the highest observed implementation level (area C_1_) and the value of eliminating the implementation delay and gaining a perfect level of implementation (area C_2_). The value of eliminating implementation delay presented here assumes that the implementation patterns would be the same and that they are simply moved back to an earlier starting point. For simplicity and ease of graphical representation, this work assumes that the value of eliminating implementation delay is captured in the period until implementation starts rather than over time, as a parallel shift in the implementation curve would entail. The undiscounted results are the same, but with a positive discount rate, this would lead to a slight overestimation. Each of these subcategories of pEVPIM represents the expected value of different specific implementation improvements (i.e., pEVSIM), as further detailed in the equations outlined in Supplemental Table S1.

This subcategorization identifies the absolute gains of eliminating slow, low, and delayed implementation, as well as what proportion of pEVPIM that is related to each of these parts of suboptimal implementation. Combined, the pEVSIM of eliminating slow, low, and delayed implementation (areas A, B, and C) make up the pEVPIM(i.e., the value of achieving *instant and full* implementation), as Andronis and Barton^7^ described pEVPIM. In this work, we use the terms *perfect level of implementation* to represent “full” implementation in all eligible patients and *perfect implementation* as instantaneous achievement of such a perfect level of implementation.

### Regional Implementation Variation

A further dimension to consider is implementation variation across different health care entities (i.e., health care regions, hospitals, health care clinics, or similar). Implementation variation is a major challenge for health care systems and was an important driver in the development of the National Institute for Health and Care Excellence (NICE), set up to overcome the so-called postcode lottery in England.^20,21^ By incorporating implementation variation in the subcategorization of EVPIM, we can estimate the proportion of EVPIM that is related to implementation variation.

We define the value of eliminating implementation variation (area D) as the value of increasing implementation up to the level of the highest implementing entity ($\rho_{t}^{\mathrm{high}}$) in each time period, from the start of implementation (Figure 1c). The value of eliminating implementation variation comes from reducing slow and/or low implementation in those health care entities that have a lower level of implementation than the highest implementing entity. Unless the highest implementing entity has achieved perfect implementation, there will still be value in addressing additional slow and low implementation compared to the implementation in the highest implementing entity. These values are here defined as the value of eliminating additional slow implementation (area E) and the value of eliminating additional low implementation (area F), compared to the highest implementing entity, as outlined in Figure 1c. The value of eliminating delayed implementation (area C) remains the same as previously outlined, but C_2_ is further divided into C_2a_, representing the value of eliminating delay as well as increasing implementation to the highest observed implementation level ($\rho_{t}^{\mathrm{high}}$), and C_2b_, the value of eliminating delay as well as increasing implementation to the perfect level of implementation.

This subcategorization shows the absolute and relative value of addressing regional implementation variation compared to eliminating slow, low, or delayed implementation. There may be reasons for not benchmarking against the level of the single highest implementing entity. Instead, $\rho_{t}^{\mathrm{high}}$ can be defined as the level of implementation in the top 3, top 5, or top 50% of implementing entities, as illustrated in the case study.

### Case Study

We illustrate the EVPIM subcategorization outlined above using a case study from cardiovascular disease. The case study is based on the implementation of the oral P2Y12 inhibitor ticagrelor for secondary prevention after MI in Sweden.^22–25^ Ethical approval for this case study was granted from the regional ethics board in Linköping, Sweden (Regionala etikprövningsnämnden i Linköping; Dnr 2018/26-31).

Ticagrelor was approved by the European Medicines Agency (EMA) on December 3, 2010, for “prevention of atherothrombotic events in adult patients with Acute Coronary Syndromes.”^26^ The Swedish Dental and Pharmaceutical Benefits Board (Tandvård- och läkemedelsförmånsverket [TLV]) deemed ticagrelor to be cost-effective and granted it reimbursement on June 9, 2011, and ticagrelor was included in the national treatment guidelines by the National Board of Health and Welfare (Socialstyrelsen) on December 21, 2011.^27,28^ (Ticagrelor was later also approved for use in other indications, but this analysis focuses on the implementation within the first approved treatment indication in secondary prevention of MI.)

Data on ticagrelor effectiveness and cost-effectiveness for this case study were sourced from a published cost-effectiveness assessment of ticagrelor in a Swedish setting, which presented a long-term cost-effectiveness assessment based on the Study of Platelet Inhibition and Patient Outcomes (PLATO) trial (Table 1).^25^ For the estimation of INB in the present case study, a cost-effectiveness threshold of SEK 250,000 (approximately £22,300) per quality-adjusted life year (QALY) was used, a conservative figure compared to the threshold employed by TLV in the assessment of similar technologies.^29^ The discount rate was set at 3% per year in accordance with TLV’s guidelines on economic evaluations.^30,31^

**Table 1:** Input to the Ticagrelor Case Study

	Ticagrelor	Clopidogrel	Δ
Life years^25^	11.47	11.32	0.15
QALY^25^	9.66	9.53	0.13
Total health care costs in SEK^25^	346,803 (£30,935)	343,560 (£30,646)	3243 (£289)
ICER in SEK/QALY (£/QALY)^25^			25,022 (£2232)
INHB per patient in QALY^a^			0.117
INMB per patient in SEK (£)^a^			29,257 (£2610)

ICER, incremental cost-effectiveness ratio; INHB, incremental net health benefit; INMB, incremental net monetary benefit; QALY, quality-adjusted life year.

a Based on a threshold of SEK 250,000 (£22,300).

**Figure 2:**
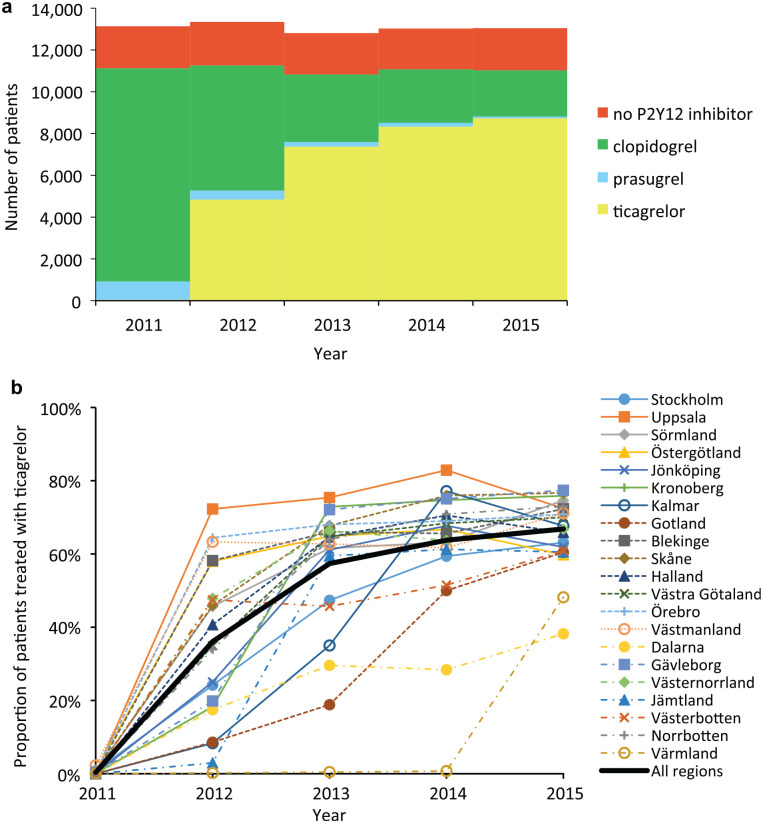
(a) Number of MI patients (age<80) with and without P2Y12 inhibitor in Sweden; and (b) Proportion of dual antoplatelet treated MI patients (age<80) receiving ticagrelor per health care region in Sweden.

Estimation of ticagrelor implementation levels was based on utilization data from the Swedish Web System for Enhancement and Development of Evidence-Based Care in Heart Disease Evaluated According to Recommended Therapies (SWEDEHEART) registry. A full description of SWEDEHEART is available elsewhere.^32,33^ From this nationwide registry, the number of patients (age <80 years) treated with ticagrelor, clopidogrel, prasugrel, or no P2Y12 inhibitor was obtained per health care region (county council), as shown in Figure 2. Focusing on patients age <80 years is consistent with Swedish and international guidelines recommending ticagrelor in this patient population.^28,34^

We defined the number of eligible patients as the number of patients who received dual antiplatelet treatment (i.e., we excluded patients who received clopidogrel or acetylsalicylic acid (ASA) as monotherapy in the main analysis). Hence, the level of ticagrelor implementation is estimated as the proportion of dual antiplatelet-treated patients who received ticagrelor and ASA. Based on this definition, the highest (average) level of implementation (*max*(ρ_*t*_)) was identified to be 79%, and the highest level achieved in a specific health care region (*max*($\rho_{t}^{\mathrm{high}}$)) was 94%.

Prasugrel is another dual antiplatelet that was approved by the EMA and reimbursed in Sweden by TLV prior to the approval of ticagrelor. There was limited use of prasugrel in Sweden during 2010 and after the approval of ticagrelor (as seen in Suppl. Figure S1). However, to avoid overestimating the value of improving ticagrelor implementation, the estimations were adjusted for the observed prasugrel utilization by subtracting the number of patients treated with prasugrel from the number of additional patients treated with ticagrelor under improved implementation (in effect assuming similar INB from ticagrelor and prasugrel treatment).

#### Scenario analysis

We also assessed the value of increasing implementation in some of the patients who did not receive dual antiplatelet treatment. The SWEDEHEART data showed that the proportion of patients not receiving dual antiplatelet treatment varied significantly between regions (from around 5%–25%) (Suppl. Figure S2). In this scenario analysis, we estimated the value of increasing implementation up to the implementation level in the health care region with the lowest proportion of nontreated patients. The value of increasing implementation in this patient population was estimated given different assumptions on INB: 1) same effect and INB as estimated from the PLATO trial, 2) double QALY gain and same cost as estimated form the PLATO trial, 3) half the QALY gain and same cost as estimated from the PLATO trial, and 4) zero QALY gain but same cost as estimated for the PLATO trial.

We also conducted different sensitivity analyses on the threshold, treatment effect, and cost of treatment.

### Funding

Financial support for this study was provided in part by a grant from Östergötland Region, Sweden. The funding agreement ensured the authors’ independence in designing the study, interpreting the data, writing, and publishing the results.

## Results from the Case Study

The pEVPIM is estimated to 2941 QALYs or SEK 735 million (£66 million) in this case study of ticagrelor implementation (Table 2).

**Table 2:** Results from the Ticagrelor Case Study^a^

	2011	2012	2013	2014	2015	Total	% of pEVPIM
pINB	6	550	813	892	908	3168	
pEVPIM	1297	730	381	295	239	2941	
Value of eliminating slow, low, and delayed implementation							
A	0	463	133	48	0	644	21.9
B	0	266	249	247	239	1000	34.0
C	1297	0	0	0	0	1297	44.1
C1	1026	0	0	0	0	1026	34.9
C2	271	0	0	0	0	271	9.2
Value of eliminating regional implementation variation							
D	26	504	232	208	167	1138	38.7
E	0	146	75	13	0	234	8.0
F	0	79	74	74	71	298	10.1
C1	1026	0	0	0	0	1026	34.9
C2a	190	0	0	0	0	190	6.5
C2b	55	0	0	0	0	55	1.9
Sensitivity analysis							
Increased implementation level up to							
Highest implementing region (D)	26	504	232	208	167	1138	38.7
Top 3 regions	13	446	215	187	126	989	33.6
Top 5 regions	9	418	198	171	99	896	30.5
Top 10 regions	3	282	162	132	68	647	22.0
Different assumptions on effect from increasing proportion receiving dual antiplatelet therapy							
1) Same effect and INB as estimated from the PLATO trial						505	
2) Double QALY gain and same cost as estimated form the PLATO trial						1066	
3) Half the QALY gain and same cost as estimated from the PLATO trial						225	
4) Zero QALY gain but same cost as estimated for the PLATO trial						−56	

EVPIM, expected value of perfect implementation; PLATO, Platelet Inhibition and Patient Outcomes.

a Data show incremental net benefit estimates (INBs) in quality-adjusted life years (QALYs) from eliminating slow (A), low (B), and delayed (C = C1 + C2) implementation, as well as the value of eliminating regional implementation variation (D).

### Slow, Low, and Delayed Implementation Subcategorization

The results demonstrate that the value of eliminating slow implementation represents 22% of the pEVPIM, and low and delayed implementation represent 34% and 44%, respectively (Table 2). This indicates that the value of eliminating low implementation compared to slow implementation is around 50% higher in the case of ticagrelor implementation in Sweden.

**Figure 3:**
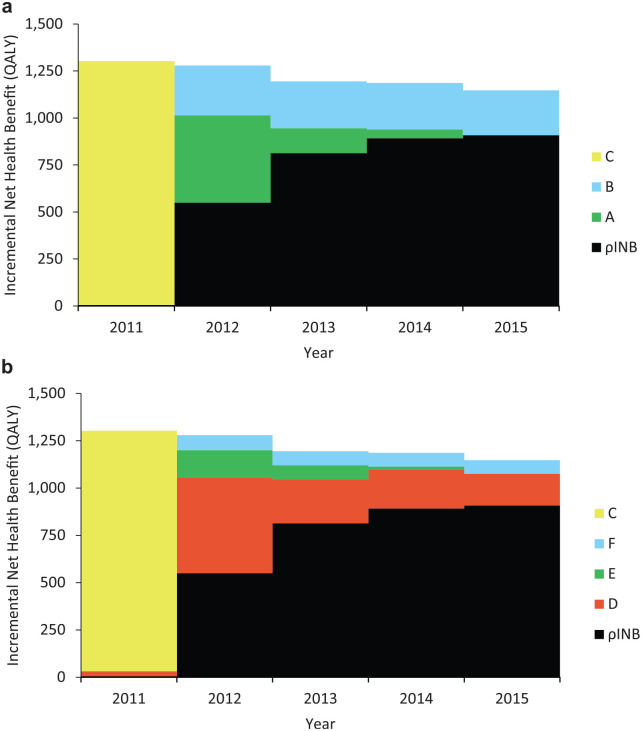
The incremental net health benefit from (a) eliminating slow (A), low (B) and delayed (C) implementation; and (b) eliminating regional implementation variation (D), based on the ticagrelor case study.

### Regional Implementation Variation

In terms of regional variation, it is estimated that 39% of pEVPIM, or 1138 QALYs, would have been gained from eliminating regional variation. The value from further eliminating slow and low implementation is reduced compared to the previous subcategorization, since the majority of this value is incorporated in the value of eliminating regional implementation variation.

The sensitivity analyses demonstrated that the value of eliminating regional implementation variation was 989 QALYs and 34% of the pEVPIM when estimating the value of increasing implementation up to the average implementation in the 3 highest implementing regions. This value was reduced to 647 QALYs and 22% of the pEVPIM when increasing the implementation of the bottom 50% up to the average implementation level of the 50% of health care regions with the highest level of implementation (Table 2).

### Sensitivity Analysis

It was estimated that 4575 additional patients would have been treated with dual antiplatelets if all regions had treated the same proportion of patients as the regions with the highest proportion of dual antiplatelet treatment. The estimated value of treating these additional patients with ticagrelor varied from –56 to 1066 QALYs, depending on assumptions around the treatment effect and costs of treating these additional patients (Table 2).

Sensitivity analyses did not show any significant impact on the results presented above for this case study. The absolute INB estimations were, as expected, affected by different assumptions on cost, effects, and threshold value, but the proportion of pEVSIM related to slow, low, delayed or varying implementation remained similar across the scenario analysis.

## Discussion

### Main Findings from the Case Study

The case study of the implementation of ticagrelor demonstrates how the subcategorization of EVPIM can be applied to estimate the value of eliminating slow, low, delayed, or varying implementation and thus provides an upper limit for how much could have been gained from eliminating each of these parts of suboptimal implementation. In this case study, 39% of the pEVPIM, representing 1138 QALYs, could potentially have been gained by eliminating regional implementation variation. In fact, in the period after implementation was initiated, regional variation accounted for around 70% of the health loss due to suboptimal implementation. This demonstrates that there can be significant health gains from eliminating implementation variation, even in a relatively small country where equal access to health care is an explicit objective.^35–37^

Notably, this case study is based on retrospective implementation patterns. Hence, the EVPIM and EVSIM estimates represent the loss from suboptimal implementation rather than prospective value that can be obtained from improving implementation. Applying the proposed subcategorization to predictions of future implementation patterns as well as other retrospective implementation patterns would increase our general knowledge on when and where there may be greatest returns from addressing suboptimal implementation, as discussed further below. Such general knowledge on implementation patterns may include whether regional implementation variation is a similar concern across other treatments and therapeutic areas and if this may be used to predict future, amendable, health loss from regional implementation variation across Sweden. It should be noted that the pEVSIM from eliminating regional variation was 167 QALYs (representing around SEK 41.8 million and £3.8 million) in the fifth year after implementation, indicating an upper limit to how much it could be worth (per year) to eliminate regional variation of ticagrelor treatment in later years.

In this case study, the value of eliminating delayed implementation is estimated at 1297 QALYs, representing 44% of the pEVPIM. Around half of this value occurred in the time until TLV granted ticagrelor reimbursement (June 2011) and the remaining half in the period from the reimbursement decision until the national clinical guidelines were updated and implementation started (December 2011). We do not know if updating the guidelines earlier would have resulted in earlier implementation, but the results indicate that there may be significant gains from ensuring that treatment guidelines are updated at the same time or as part of the reimbursement/HTA process, similar to the NICE processes in England. It is, however, important to stress that our results do not imply that the time until HTA decision should be minimized to reduce the potential loss due to implementation delay. There are good reasons for having a rigorous HTA and cost-effectiveness assessment of new health care technologies to ensure that only those technologies that are expected to provide a positive INB are implemented, as detailed by, for example, Johannesen et al.^38^

A key challenge in all EVPIM studies, and ours is no exception, is to define the number of eligible patients. In this work, we defined eligible patients as all MI patients who received dual antiplatelet therapy. In this way, the case study incorporates clinicians’ assessment of which patients should receive dual antiplatelet treatment and potential variation in case mix across health care regions. This estimate is conservative compared with using all MI patients indicated to receive dual antiplatelet therapy as an estimate of eligible patients. The large variation in the proportion of patients treated with dual antiplatelet therapy across regions, nevertheless, indicates that further investigation may be warranted to understand if this variation is due to different case mix and/or varying treatment practice of MI patients across Swedish health care regions.

The benefits from improving implementation in this case study rely on a cost-effectiveness assessment based on the PLATO trial and assume the same INB per patient treated as derived from this study. A registry study of MI patients treated with ticagrelor as secondary prevention in Sweden (*N* = 45,073, with 11,954 patients treated with ticagrelor) found similar effectiveness of ticagrelor treatment as the PLATO trial,^39^ supporting the approach of this case study. Neither the PLATO trial nor the study by Sahlén et al.^39^ provided evidence on significant effectiveness or cost variations for the patient population of the case study.^22,25^ This supports the assumption of constant INB per patient treated. The issue of heterogeneous INB is discussed further below.

### Strength and Limitations of the Proposed Subcategorization of EVPIM

A benefit of the proposed framework of subcategorizing EVPIM is that it identifies the value of addressing different parts of suboptimal implementation, similar to EVPPI in the EVPI framework.^5,6^ By disentangling the value of addressing slow, low, delayed, and varying implementation from the total EVPIM, assessment of implementation strategies addressing these different parts is facilitated. This approach also enables evaluation of the value of implementation strategies that address specific parts of suboptimal implementation across several health care interventions or therapeutic areas (e.g. strategies that could reduce regional variation across several interventions).

The categories of slow, low, and delayed implementation were selected as they represent different aspects of suboptimal implementation that potentially require different strategies to address them. Slow implementation represents the time it takes to reach the highest achieved/expected level of implementation (i.e., the loss from not getting to this level faster). In contrast, low implementation relates to the loss from the achieved/expected level of implementation being lower than the optimal level of implementation (optimal level of implementation could be defined by clinical experts, guidelines, or HTA agencies). Delayed implementation represented a loss from not starting implementation earlier, which is likely linked to policy processes such as updating of treatment guidelines, inclusion on drug formularies, and securing funding for new therapies. Further understanding and research into how different implementation strategies address slow, low, and delayed implementation are needed to determine the relevance of this subcategorization, as well as understand if there are other more relevant subcategorizations.

When there is significant implementation variation, there is an opportunity to understand what higher implementing regions, hospitals, or physicians are doing differently and aim to establish how this might be used by others. Indeed, a key reason for the development and expansion of clinical and quality registries in recent years has been to identify and understand clinical practice variations.^40^ The approach presented in this article could help to assess the consequences of observed/expected implementation variations, estimate the value of reducing variations, and help to identify patterns associated with the highest value of being improved.

The approach of subcategorizing EVPIM can be applied to retrospective implementation patterns, as in the current case study, as well as future predicted implementation patterns. Works by Grimm et al.^13–15^ have outlined methods for predicting future implementation patterns, based on diffusion theory and expert beliefs. Although it is not yet common practice to perform detailed predictions of future implementations patterns, this work demonstrates how EVPIM and EVSIM, as well as our proposed subcategorization, can be estimated prior to the introduction of new technologies.

Applications of the proposed subcategorizations to past implementation patterns might be equally valuable for understanding how to address the issue of suboptimal implementation. Applying this framework broadly to previously implemented technologies could identify the relative size of slow, low, delayed, and varying implementation out of pEVPIM. In addition to identifying what has been the main contributor(s) of pEVPIM, this information could identify how slow, low, delayed, and varying implementation differ across therapeutic areas, types of health care technologies, geographic areas, and physician specialty. Such analyses would be highly valuable to identify areas most in need of implementation strategies and to indicate what type of future implementation research and strategies to investigate and employ.

A key limitation to estimating the value of eliminating slow, low, or varying implementation, as well as overall EVPIM, across health care technologies is the availability of implementation patterns, defining the number of eligible patients and cost-effectiveness estimates. Case studies, including ours, tend to focus on pharmaceuticals or medical devices, most likely due to the availability of published data and clearly defined patient populations. However, as exemplified by Hoomans and colleagues,^41–44^ who estimated the value of guideline implementation, and Mewes et al.,^45^ who evaluated the value of increasing adherence to guideline-based physical exercise for cancer survivors, it is possible to apply the EVPIM framework to other and broader implementation assessments. Work has also shown how heterogeneous cost-effectiveness estimates may be addressed in the EVPIM framework^10,11^ and the effect of nonlinear costs (e.g., cases where investment in new equipment is needed) and nonlinear effect (e.g., herd immunity from vaccinations) could also be accounted for in EVPIM estimations. It may be more challenging to clearly define the time of availability for surgical procedures and changes in treatment practices compared to pharmaceuticals and medical devices, but first adoption into routine use and recommendations by clinical societies, clinical guidelines, or similar could be used as alternatives for EMA or HTA recommendations.

### Policy Implications

Low implementation and unequal access to health care are key hurdles for health care systems around the world.^1,2^ Subcategorizing EVPIM could provide valuable information to policy and decision makers on how to address these issues through identifying 1) what parts of suboptimal implementation are contributing most to pEVPIM, 2) the value of eliminating different parts of suboptimal implementation, and 3) regions, health care providers, and therapeutic areas where there is highest value from improving implementation.

EVPIM subcategorization provides a way to identify and guide research and investments in implementation strategies for those parts of suboptimal implementation that are causing the greatest loss to population health. Applying the proposed subcategorizing across health care technologies could help to assess the value of implementation strategies that address different parts of suboptimal implementation across multiple health care technologies and therapeutic areas. In this way, EVPIM subcategorization could support assessment and prioritization between implementation strategies and help decision makers to identify what implementation strategies to fund.

## Supplemental Material

MDM_supplementary_file_-_20_Jan_2020.rjf_online_supp – Supplemental material for Subcategorizing the Expected Value of Perfect Implementation to Identify When and Where to Invest in Implementation Initiatives Click here for additional data file. Supplemental material, MDM_supplementary_file_-_20_Jan_2020.rjf_online_supp for Subcategorizing the Expected Value of Perfect Implementation to Identify When and Where to Invest in Implementation Initiatives by Kasper Johannesen, Magnus Janzon, Tomas Jernberg and Martin Henriksson in Medical Decision Making
