# Supplementary material for: Subcategorizing the Expected Value of Perfect Implementation to Identify When and Where to Invest in Implementation Initiatives
Source: Med Decis Making. 2020 Mar 5;40(3):327–38. doi: 10.1177/0272989X20907353 (PMC7488812; doi:10.1177/0272989X20907353)
Supplement: MDM_supplementary_file_-_20_Jan_2020.rjf_online_supp – Supplemental material for Subcategorizing the Expected Value of Perfect Implementation to Identify When and Where to Invest in Implementation Initiatives [file MDM_supplementary_file_-_20_Jan_2020.rjf_online_supp.docx]

# Supplementary Materials

*Figure S1 Proportion of MI patients (age<80) receiving prasugrel by Swedish county councils.*

*Figure S2 Proportion of MI patients (age<80) receiving no P2Y12 inhibitor by Swedish county councils.*

*Figure S3 Proportion of MI patients (age<80) receiving clopidogrel by Swedish county councils.*

Table S1 Definitions of the Value of Eliminating Slow, Low, Delayed and Varying Implementation

| **Description** | **Area** | **Equation** |
| --- | --- | --- |
| Population INB |  | $\boldsymbol{p}\text{INB}\text{=}\sum_{\boldsymbol{t}\boldsymbol{=}\boldsymbol{0}}^{\boldsymbol{T}} \frac{\boldsymbol{I}_{\boldsymbol{t}}}{\boldsymbol{(1+r)}^{\boldsymbol{t}}}\text{ρ}_{\boldsymbol{t}}\text{INB}_{\boldsymbol{t}}$ |
| **Slow, low and delayed implementation** | | |
| Population EVPIM |  | $\boldsymbol{p}\text{EVPIM}\text{=}\sum_{\boldsymbol{t=0}}^{\boldsymbol{T}} \frac{\boldsymbol{I}_{\boldsymbol{t}}}{{\boldsymbol{(}\boldsymbol{1}\boldsymbol{+}\boldsymbol{r}\boldsymbol{)}}^{\boldsymbol{t}}}\boldsymbol{(}\boldsymbol{1}\boldsymbol{-}\text{ρ}_{\boldsymbol{t}}\boldsymbol{)}\text{INB}_{\boldsymbol{t}}$ |
| **Eliminating slow implementation**, i.e. implement up to *max*(ρ*_t_*) from the time when implementation starts (*t_implement_*) | A | ${\boldsymbol{p}\mathbf{EVSIM}}_{\mathbf{A}}\text{=}\sum_{{\boldsymbol{t}\boldsymbol{=}\boldsymbol{t}}_{\boldsymbol{implement}}}^{\boldsymbol{T}} \frac{\boldsymbol{I}_{\boldsymbol{t}}}{\boldsymbol{(1+r)}^{\boldsymbol{t}}}\boldsymbol{(}\boldsymbol{max}\boldsymbol{(}\text{ρ}_{\boldsymbol{t}}\boldsymbol{)-}\text{ρ}_{\boldsymbol{t}}\boldsymbol{)}\text{INB}_{\boldsymbol{t}}$ |
| **Eliminating low implementation**, i.e. implement in 100%-*max*(ρ*_t_*) from *t_implement_* | B | ${\boldsymbol{p}\mathbf{EVSIM}}_{\mathbf{B}}\text{=}\sum_{\boldsymbol{t}\boldsymbol{=}\boldsymbol{t}_{\boldsymbol{implement}}}^{\boldsymbol{T}} \frac{\boldsymbol{I}_{\boldsymbol{t}}}{\boldsymbol{(1+r)}^{\boldsymbol{t}}}\boldsymbol{(}\boldsymbol{1}\boldsymbol{-}\boldsymbol{max}\boldsymbol{(}\text{ρ}_{\boldsymbol{t}}\boldsymbol{))}\text{INB}_{\boldsymbol{t}}$ |
| **Eliminating implementation delay**, i.e. implement from the time of availability (*t_0_*) rather than time *t_implement_* | C | $\boldsymbol{p}\mathbf{EVSIM}_{\mathbf{C}}\text{=}\sum_{\boldsymbol{t}\boldsymbol{=}\boldsymbol{0}}^{\boldsymbol{t}_{\boldsymbol{implement}}} \frac{\boldsymbol{I}_{\boldsymbol{t}}}{\boldsymbol{(1+r)}^{\boldsymbol{t}}}\text{INB}_{\boldsymbol{t}}$ |
| Eliminating implementation delay given actual/expected implementation pattern | C_1_ | ${\boldsymbol{p}\mathbf{EVSIM}}_{\mathbf{C}_{\mathbf{1}}}\text{=}\sum_{\boldsymbol{t}\boldsymbol{=}\boldsymbol{0}}^{\boldsymbol{t}_{\boldsymbol{implement}}} \frac{\boldsymbol{I}_{\boldsymbol{t}}}{\boldsymbol{(1+r)}^{\boldsymbol{t}}}\boldsymbol{max}\boldsymbol{(}\text{ρ}_{\boldsymbol{t}}\boldsymbol{)}\text{INB}_{\boldsymbol{t}}$ |
| Eliminating implementation delay in 100%-*max*(ρ*_t_*) | C_2_ | ${\boldsymbol{p}\mathbf{EVSIM}}_{\mathbf{C}_{\mathbf{2}}}\text{=}\sum_{\boldsymbol{t}\boldsymbol{=}\boldsymbol{0}}^{\boldsymbol{t}_{\boldsymbol{implement}}} \frac{\boldsymbol{I}_{\boldsymbol{t}}}{\boldsymbol{(1+r)}^{\boldsymbol{t}}}{\text{(1-}\boldsymbol{max}\boldsymbol{(}\text{ρ}_{\boldsymbol{t}}\boldsymbol{))}\text{INB}}_{\boldsymbol{t}}$ |
| **Accounting for regional implementation variation** | | |
| **Eliminating regional variation in implementation**, i.e. implement in $\rho_{t}^{hight}$-ρ*_t_* from *t_implement_* | D | ${\boldsymbol{p}\mathbf{EVSIM}}_{\mathbf{D}}\text{=}\sum_{\boldsymbol{t}\boldsymbol{=}\boldsymbol{t}_{\boldsymbol{implement}}}^{\boldsymbol{T}} \frac{\boldsymbol{I}_{\boldsymbol{t}}}{\left( \boldsymbol{1}\boldsymbol{+}\boldsymbol{r} \right)^{\boldsymbol{t}}}\boldsymbol{(}\boldsymbol{\rho}_{\boldsymbol{t}}^{\boldsymbol{high}}\boldsymbol{-}\text{ρ}_{\boldsymbol{t}}\boldsymbol{)}\text{INB}_{\boldsymbol{t}}$ |
| Eliminating slow implementation compared to implementation in highest implementing region, i.e. implement in *max*($\rho_{t}^{hight}$)-($\rho_{t}^{hight}$) from t_implement_ | E | ${\boldsymbol{p}\mathbf{EVSIM}}_{\mathbf{E}}\text{=}\sum_{\boldsymbol{t}\boldsymbol{=}\boldsymbol{t}_{\boldsymbol{implement}}}^{\boldsymbol{T}} \frac{\boldsymbol{I}_{\boldsymbol{t}}}{\left( \boldsymbol{1+r} \right)^{\boldsymbol{t}}}\boldsymbol{(}\boldsymbol{max}\left( \boldsymbol{\rho}_{\boldsymbol{t}}^{\boldsymbol{high}} \right)\boldsymbol{-}\boldsymbol{\rho}_{\boldsymbol{t}}^{\boldsymbol{high}}\boldsymbol{)}\text{INB}_{\boldsymbol{t}}$ |
| Eliminating low implementation compared to highest observed implementation level, i.e. implemented in 100%-*max*($\rho_{t}^{hight}$) from t_implement_ | F | $\boldsymbol{p}\mathbf{EVSIM}_{\mathbf{F}}\text{=}\sum_{{\boldsymbol{t}\boldsymbol{=}\boldsymbol{t}}_{\boldsymbol{implement}}}^{\boldsymbol{T}} \frac{\boldsymbol{I}_{\boldsymbol{t}}}{\left( \boldsymbol{1+r} \right)^{\boldsymbol{t}}}\boldsymbol{(}\boldsymbol{1}\boldsymbol{-}\boldsymbol{max}\left( \boldsymbol{\rho}_{\boldsymbol{t}}^{\boldsymbol{high}} \right)\boldsymbol{)}\text{INB}_{\boldsymbol{t}}$ |
| Eliminating implementation delay given implementation pattern in the highest implementing region | C_2a_ | ${\boldsymbol{p}\mathbf{EVSIM}}_{\mathbf{C}_{\mathbf{2a}}}\text{=}\sum_{\boldsymbol{t}\boldsymbol{=}\boldsymbol{0}}^{\boldsymbol{t}_{\boldsymbol{implement}}} \frac{\boldsymbol{I}_{\boldsymbol{t}}}{\left( \boldsymbol{1+r} \right)^{\boldsymbol{t}}}\boldsymbol{(}\boldsymbol{max}\left( \boldsymbol{\rho}_{\boldsymbol{t}}^{\boldsymbol{high}} \right)\boldsymbol{-}\boldsymbol{max}\left( \text{ρ}_{\boldsymbol{t}} \right)\boldsymbol{)}\text{INB}_{\boldsymbol{t}}$ |
| Eliminating implementation delay in 100%-*max*($\rho_{t}^{hight}$) | C_2b_ | $\boldsymbol{p}\mathbf{EVSIM}_{\mathbf{C}_{\mathbf{2a}}}\text{=}\sum_{\boldsymbol{t}\boldsymbol{=}\boldsymbol{0}}^{\boldsymbol{t}_{\boldsymbol{implement}}} \frac{\boldsymbol{I}_{\boldsymbol{t}}}{\boldsymbol{(1+r)}^{\boldsymbol{t}}}\boldsymbol{(}\boldsymbol{1}\boldsymbol{-}\boldsymbol{max}\left( \boldsymbol{\rho}_{\boldsymbol{t}}^{\boldsymbol{high}} \right)\boldsymbol{)}\text{INB}_{\boldsymbol{t}}$ |
| INB*_t_* is the expected incremental net benefit defined as the expectation over some uncertain parameters θ (E_θ_INB(θ)) at time t, which can be estimated in terms of incremental net health benefit (INHB) or incremental net monetary benefit (INMB): $INHB=\Delta E-\Delta C/\lambda; INMB=\Delta E*\lambda-\Delta C$, where ΔE and ΔC is the incremental effect and cost, respectively, and λ is the cost-effectiveness threshold  $I_{t}$ total number of eligible patients in time period t  *r* is the discount rate  ρ*_t_* is the actual/expected level of implementation in time period t  *max*(ρ*_t_*) is the highest (average) level of implementation observed across all time periods  $\rho_{t}^{high}$ is the implementation level in the **highest implementing region** at time t  *max*($\rho_{t}^{high}$) is the highest level of implementation observed in any region across all time periods  *T* is the time at which the intervention loses relevance  *t_0_* is the time when the technology becomes available for use  *t_implement_* is the time at where implementation starts | | |
